# Biotransformations with Photobiocatalysts for Enantioselective Ester Hydrolysis

**DOI:** 10.3390/molecules30132767

**Published:** 2025-06-27

**Authors:** Agnieszka Śliżewska, Paulina Majewska, Ewa Żymańczyk-Duda

**Affiliations:** Department of Biochemistry, Molecular Biology and Biotechnology, Faculty of Chemistry, Wrocław University of Science and Technology, Wybrzeże Stanisława Wyspiańskiego 27, 50-370 Wrocław, Poland; majewska.p@gmail.com

**Keywords:** biotransformation, cyanobacteria, photobiocatalysts, hydrolysis

## Abstract

This study investigates the efficient and enantioselective hydrolysis of ester bonds through a series of biotransformations employing various photobiocatalysts. A racemic mixture of 1-phenylethyl acetate served as the model substrate. The described research identified three strains exhibiting the highest biocatalytic activity: *Nostoc cf-muscorum* (CCALA 129), *Leptolyngbya foveolarum* (CCALA 76), and *Synechococcus bigranulatus* (CCALA 187). Their application led to the complete hydrolysis of the starting reagent, yielding both the unreacted ester and its corresponding alcohol in an enantioselective manner. Notably, the selectivity, expressed as S, reached an impressive value of 283 in certain outcomes. The photobiotransformations were conducted under varying conditions, with particular focus on two essential parameters: the duration of the process, crucial for kinetically controlled reactions, and light exposure, critical for light-dependent organisms. The representative results highlight the efficacy of these biocatalysts. For instance, using *Leptolyngbya foveolarum* (CCALA 76), *Nostoc cf-muscorum* (CCALA 129), and *Synechococcus bigranulatus* (CCALA 187) facilitated the production of 1-(*R*)-phenylethanol with enantiomeric excesses (ee) of 89%, 88%, and 86%, respectively, at a conversion degree of approximately 50%. These processes also yielded an optically enriched mixture of the unreacted substrate, 1-(*S*)-phenylethyl acetate. Specifically, in the case of *Leptolyngbya foveolarum* (CCALA 76), the ee of the unreacted ester reached up to 98%. Light exposure emerged as a key factor influencing selectivity factor (S). Adjusting this parameter allowed us to achieve an *E* value of up to 283 for the formation of 1-(*R*)-phenylethanol with an ee > 99% when utilizing the *Nostoc cf-muscorum* (CCALA 129) strain. Furthermore, light intensity proved crucial for scaling up these processes. Significant results were obtained with *Synechococcus bigranulatus*, particularly at substrate concentrations ranging from 1 to 10 mM under limited exposure. Here, the conversion degree was 55%, the ee of the (*R*)-alcohol was 86%, and the selectivity factor (S) value was 21.

## 1. Introduction

Cyanobacteria, a highly diverse group of prokaryotes, are ubiquitous across virtually all ecological niches and are fundamentally dependent on oxygenic photosynthesis [1]. Their inherent susceptibility to genetic recombination renders them highly valuable for industrial applications, particularly in the production of high-value compounds. A notable example is the application of a recombinant *Synechocystis* sp. *PCC6803* strain, into which prokaryotic enoate reductase (YqjM) genes were incorporated. This strain facilitated the asymmetric reduction of C=C bonds, yielding optically pure 2-methylsuccinimide with 99% enantiomeric excess (ee) and an 80% isolated product yield [2]. Furthermore, engineered cyanobacterial strains have demonstrated the capacity for the highly efficient production of trehalose, a saccharide extensively utilized across the food, cosmetics, and pharmaceutical sectors [3]. Despite their demonstrated utility, the comprehensive potential of cyanobacteria remains largely unexplored. Of the 376 species documented in the CyanoBase database, only 86 genomes have been fully characterized [4]. Given that only an estimated 1% of microalgae species are currently known, continued research into these organisms is of paramount importance [5]. Cyanobacteria also represent a rich reservoir of compounds exhibiting significant biological activity, primarily synthesized as secondary metabolites. Many of these possess advantageous properties, such as antiviral or antibacterial characteristics, making them attractive candidates for the pharmaceutical industry. This group encompasses diverse chemical classes, including alkaloids, peptides, and polyketides [6]. Conversely, some secondary metabolites are inherently toxic, a characteristic often linked to the adaptive survival strategies of these organisms in varied environments. Such toxins can pose considerable health hazards to humans and animals and exert negative impacts on ecosystems. Among the most extensively studied are microcystins, anatoxins, and nodularins [7]. Beyond their direct metabolic products, cyanobacterial biomass serves as a versatile resource for dietary supplements, polymer and biofuel production, bioplastics, and wastewater treatment, among numerous other applications [8]. Their contribution to industrial enzymes alone is projected to reach an estimated value of USD 7.0 billion by 2023 [9]. Significantly, these microorganisms are effectively employed in whole-cell biotransformations, thereby obviating the necessity for laborious enzyme isolation [10].

The ongoing pursuit of efficient asymmetric synthesis candidates is strongly justified by industrial demands. For instance, an ene-reductase (ER) isolated from *Synechococcus* sp. *PCC 7942* has shown capability in reducing (*R*)-carvone and 2-methylmaleimide, yielding (*R*)-products with high optical purities (98% diastereomeric excess (de) and >99% ee, respectively) [11]. Biocatalysis, particularly the common hydrolytic activity, is increasingly vital for industry and is subject to continuous research as a pathway to obtain chiral high-value compounds via straightforward reactions such as ester hydrolysis [12]. Chiral alcohols are frequently targeted products, typically synthesized through enantioselective ketone reduction or ester hydrolysis. While limited literature exists on the photobiocatalytic synthesis of these compounds, the most prevalent approach involves ketone reduction using the *Synechococcus* sp. *PCC 7942* strain. This strain has been successfully applied to the reduction of aryl-methyl ketones, yielding corresponding (*S*)-alcohols with exceptional enantioselectivities. For example, 2′,3′,4′,5′,6′-pentafluoroacetophenone was converted to the (S)-alcohol with over 90% efficiency and >99% enantiomeric excess [13]. *Synechocystis* sp. *PCC 6803* has also been effectively utilized as a whole-cell biocatalyst for enantioselective reduction, resulting in chiral alcohols (up to 95% conversion and >99% ee). Research has also explored the influence of temperature, light, substrate, and cell concentration on the efficiency of substrate conversion [14].

Cyanobacteria are considered exemplary whole-cell biocatalysts owing to several inherent advantages [15]. These include minimal nutritional requirements, robust growth rates, physiological stability coupled with metabolic plasticity, and a broad tolerance to diverse harsh conditions [16]. In general, the whole cells are ready for immediate use, do not necessitate exogenous cofactors (in case of redox reactions), and exhibit elevated enzymatic stability due to their natural protective barriers [17]. These processes are generally characterized by high selectivity and catalytic efficiency. Furthermore, their reliance on mild process conditions, the elimination of enzyme isolation steps, and the potential for biocatalyst or biomass reuse post-reaction contribute to their status as environmentally friendly technologies [18,19]. The increasing meaning of photobiological systems for organic chemistry is shown by the variety of reactions carried out with such biocatalysts, e.g., fatty acids decarboxylation with photodecarboxylases or triglycerides two-step conversion with lipases and decarboxylases [20]. Considering the above, further exploration of photobiocatalyst potential is certainly warranted. 

## 2. Materials and Methods

### 2.1. Biocatalysts

All strains were purchased from the Culture Collection of Autotrophic Organisms (CCALA), Institute of Botany, Academy of Sciences, Trebon, Czech Republic. The cyanobacterial strains were *Limnospira maxima* (CCALA 27), *Kamptonema animale* (CCALA 138), *Leptolyngbya foveolarum* (CCALA 76), *Nodularia sphaerocarpa* (CCALA 114), *Nostoccf-muscorum* (CCALA 129), *Synechococcus bigranulatus* (CCALA 187), and *Nodularia moravica* (CCALA 797).

### 2.2. Chemicals

All chemicals were purchased from Sigma Aldrich (Poznań, Poland), Merck (Poznań, Poland), and Avantor Performance Materials (Gliwice, Poland). 

### 2.3. Cultivation Conditions

All cultures were grown in 250 mL Erlenmeyer flasks, except the strain of *Kamptonema animale* (CCALA 138), which was grown in a flat-bottom flask of the same capacity. In the process of examining the effect of the incident light surface on the efficiency of the biotransformation process, sterile cell culture bottles with 600 mL capacity were used for each culture. In every case, 100 mL of suitable medium was used to grow the culture.

Three culture media were applied for the culturing, depending on the cultivated strain. *Kamptonema animale* (CCALA 138), *Leptolyngbya foveolarum* (CCALA 76), *Nodularia sphaerocarpa* (CCALA 114), *Synechococcus bigranulatus* (CCALA 187), and *Nodularia moravica* (CCALA 797) strains were grown on BG-11 medium. The *Nostoc cf-muscorum* (CCALA 129) strain was grown on BG-11-0 without NaNO_3_, while the *Limnospira maxima* (CCALA 27) strain was grown on Spirulina Medium (SM).

The culture conditions were as follows: stationary cultivation; temperature of the breeding at (±1 °C) 30 °C; a cultivation time of 21 days (final cells density ranged from 10^6^ to 10^7^ cells/mL); with continuous illumination using a Hagen PowerGlo fluorescent lamp (T8, 30W, 18,000 K, 1330 Lumens) (Aqua-Light, Gostynin, Poland). 

### 2.4. Biotransformations Procedure

The biotransformation process started with the addition of a substrate (racemic mixture of 1-phenylethyl acetate) to a 21-day-old culture under sterile conditions. Depending on the tested strain, different concentrations of the substrate were used (ranging from 1 to 50 mM), and the process durability differed. In every case, biotransformation was stopped by the cells’ separation by centrifugation from the culture (2951× *g*, 20 min, 21 °C). The supernatant, after discarding the precipitate, was extracted twice with ethyl acetate; then, the organic layer was dried over anhydrous magnesium sulphate. After drying, the material was filtered and evaporated under reduced pressure and was analyzed.

### 2.5. Analytical Methods and GC Validation

The biotransformation products were analyzed using gas chromatography (GC). Acetophenone, 1-(*R*)-phenylethanol, and 1-(*S*)-phenylethanol were identified in comparison to commercially available standards (retention times). The ester enantiomers, 1-(*R*)-phenylethyl acetate and 1-(*S*)-phenylethyl acetate, were identified by comparison to the standards synthesized from appropriate alcohols enantiomers by esterification. Gas chromatography was performed on a Shimadzu GC-2010 Plus (Shimadzu Corporation, Duisburg, Germany), with a flame ionization detector, using a chiral column: Chirasil-Dex CB column (β-cyclodextrin) 25 m × 0.25 mm Inner Diameter (ID) × 0.25 µm film. Analyses were carried out by using nitrogen as the make-up gas in a split ratio of 10:1. The column temperature was 140 °C; the column flow was 1.2 mL/min. Here, 1μL of the sample was injected. The method validation parameters are shown in Table 1, Table 2 and Table 3. System suitability tests were performed on samples after the biotransformation process. Linearity was performed on seven different concentrations of standards. Injection precision was performed on ten injections of one concentration of standards. The accuracy and intermediate precision were calculated on three different concentrations of standards and prepared during the same day, on different days, and by different operators.

### 2.6. Cell Viability Assay

The viability of the biocatalyst cells before and after the biotransformation process was checked by Becton Dickinson FACSVerse flow cytometry (Thermo-Fisher Scientific, Warszawa, Poland). A significant problem in selecting the appropriate dye was the autofluorescence of cyanobacteria (chlorophyll). The dye SYTOX Green (Molecular Probes; Thermo-Fisher Scientific, Warszawa, Poland) was used, the fluorescence of which does not coincide with the autofluorescence of the biocatalyst. This dye only enters cells with a damaged cell membrane (dead), where it preferentially binds to DNA. Therefore, the use of this dye makes it possible to distinguish living cells from dead ones.

### 2.7. Selectivity Factor Assignment

The mixtures of the biotransformation products (alcohol and unreacted ester) were analyzed by GC using a column with chiral filling. The degree of the enantiomeric excess was expressed as a percentage (%) and defined as

(1) $$ee=\frac{P_{1}-P_{2}}{P_{1}+P_{2}}\times 100\%$$

where P_1_ and P_2_ are the values of the area values under peaks derived from enantiomers, P_1_ > P_2_. The enantiomeric ratio (*E*) was computed from the following formula:

(2) $$S=\frac{ln\left[{ee}_{p}\frac{\left(1-{ee}_{s}\right)}{\left({ee}_{s}+{ee}_{p}\right)}\right]}{ln\left[{ee}_{p}\frac{\left(1+{ee}_{s}\right)}{\left({ee}_{s}+{ee}_{p}\right)}\right]}$$

where ee_p_ is the enantiomeric excess of alcohol, and ee_s_ is the enantiomeric excess of unreacted ester. 

## 3. Results 

### 3.1. Control Tests and Substrate Stability Assignment

Control tests were carried out on 21 days of cultures without substrate addition and according to the protocols applied for bioconversions. These experiments did not reveal the presence of any compounds that could affect the biotransformation results. Additionally, the same controls were conducted on the culture media: Spirulina Medium (SM), BG-11 Medium, BG-11-0 Medium, which also excluded the presence of any additional chemical compounds, similar to the expected chemicals.

The substrate stability was confirmed with tests provided in the culture media. The substrate was added to the culture media, SM, BG-11, and BG-11-0, in a concentration of 1mM. The stability was checked under biotransformation conditions and was confirmed for the BG-11 and BG-11-0 media. For Spirulina Medium (SM), a self-active hydrolysis was observed after 3 days of the process. However, no experiments on this medium were carried out for more than 3 days. Up to 24 h, the substrate remains stable in the SM medium.

### 3.2. Screening Tests: Selection of Photobiocatalysts Active Towards Racemic 1-Phenylethyl Acetate

**Scheme 1:**
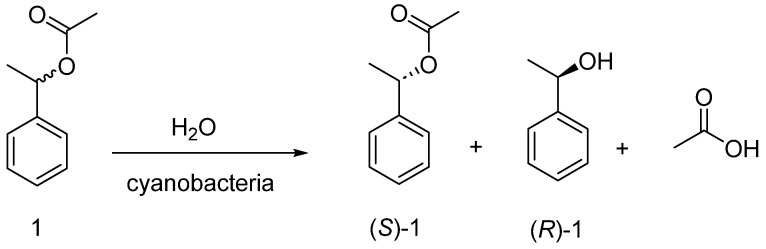
Biotransformation of racemic 1-phenylethyl acetate **1** to 1-(*R*)-phenylethanol **2** and unreacted 1-(*S*)-phenylethyl acetate with cyanobacteria assistance.

A racemic mixture of 1-phenylethyl acetate **1** was added to the 21-day-old culture of each strain to a final concentration of 1mM to determine the hydrolytic activity. The expected biotransformation is illustrated in Scheme 1. The screening photobioconversions lasted for 3 h, 1 day, and 3 days for each strain. The results are shown in Table 4. 

The above results allowed determination of the durability of the process in relation to the yield and selectivity of the reaction and led to the determination of the photobiocatalysts for further study. Thus, crucial observations were noted for *Nostoc cf-muscorum* (CCALA 129). In this case, with the time extension (from 3 to 24 h), the conversion degree increased from 50% to 74%, and the (*S*)-unreacted ester was received with the enantiomeric excess up to 99%; importantly, the selectivity achieved a higher value of 19, compared to 6.4, respectively.

The screening set also led to interesting discoveries, as further experiments proved, with an observed decrease in the alcohol concentration, in particular cases (Table 4), in connection with the competitive reaction of phenylethyl alcohol oxidation to the side product acetophenone. The oxidative course of this reaction in relation to time, was investigated in detail, as described below. 

### 3.3. Recognition of the Side Course of the Reaction: Competitive Oxidation

The reactions (Table 4), which resulted in the increase in the conversions degrees with a simultaneous decrease in the alcohol production, were analyzed to determine whether there were side processes influencing the desired outcome. Deeper analysis of the courses of the reactions allowed finding that, after ester hydrolysis, alcohol was oxidized to acetophenone. Thus, Table 5 shows the progress of this oxidation over time and for the strains with low effective alcohol production (Table 4), such as *Nodularia sphaerocarpa* or *Limnospira maxima*, where acetophenone appeared only after 24 h of bioconversions, and after 72 hrs, its concentrations reached, respectively, 12.2% and 20.2% (Table 5).

#### Acetophenone Release in Relation to Time of Oxidation for Selected Leptolyngbya Faveolarum (CCALA 76)

**Scheme 2:**
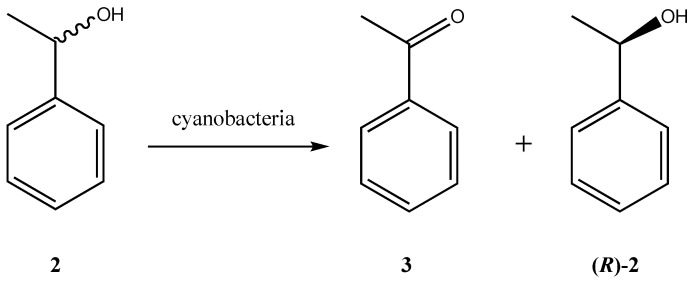
Oxidation of 1-phenylethanol to acetophenone and 1-(*R*)-phenylethanol.

The analysis of the course of oxidation in relation to the time was determined for the *Leptolyngbya foveolarum* strain, which was chosen for its stability and cultivation simplicity, compared to the others. In this case, acetophenone as a side product of bioconversion (Scheme 2) appeared after 24 h (Table 5). 

Thus, biotransformation was carried out for 1 to 10 days, according to the bioconversion protocol and was analyzed with the GC. The detected reaction products were 1-(*R*)-phenylethanol and acetophenone (at the highest concentration after 10 days). After 24 hrs of the process with *Leptolyngba faveolarum* (CCALA 76), the enantiomeric excess of the *R* alcohol achieved 39% of ee, while the conversion degree recorded for hydrolysis (reaction 1, Table 6) was 73%. This suggests that ongoing hydrolysis also resulted in the 1-(*S*)-phenylethanol formation, which was then oxidized to acetophenone, which appeared in the product mixture within the first 24 hrs. The analysis of the data in Table 6 suggests that the alcohol of *S* configuration can be the only enantiomer oxidized to acetophenone or is preferably oxidized over the *R* enantiomer. The increasing concentration of acetophenone is related to the increase in the conversion degree of hydrolysis to a final 99%. This observation is relevant to the planning of research on other hydrolysis reactions catalyzed with cyanobacteria, because it shows that achieving more than a 50% conversion degree can result in side undesired reactions.

### 3.4. Duration of Ester Bond Hydrolysis as Crucial Parameter in Kinetically Controlled Reactions

The duration of the biotransformation was adjusted individually for each of the processes and was accompanied by the simultaneous monitoring of acetophenone synthesis. Table 7 shows the results of the ester hydrolysis (1 mM of the substrate), which showed the enriched mixtures of enantiomers of unreacted substrate- 1-(*S*)-phenylethyl acetate and the product 1-(*R*)-phenylethanol in all cases. 

The *Leptolyngbya foveolarum* (CCALA 76) and *Nostoc cf-muscorum* (CCALA 129) strains turned out to have the highest activity towards the racemic mixture of 1-phenylethyl acetate used as a substrate. With a conversion degree of about 50% and releasing the alcohol enantiomer, 1- (*R*) -phenylethanol, the ee reached 89%, with good enantioselectivities for the *Leptolyngbya foveolarum* strain (CCALA 76) *E* = 30 and for *Nostoc cf-muscorum* (CCALA 129)—selectivity factor (S = 33). Simultaneously, the unreacted ester, 1-(*S*)-phenylethyl acetate, was received with moderate enantiomeric excess up to 55% and 71%, respectively (Table 7). However, hydrolysis proceeded on both enantiomers of the ester, according to the rules of the kinetic process; hence, the better the enantioselectivity the better the enantiomers resolution. The results collected in Table 7 allowed the selection of two strains of good hydrolytic activity in correlation with good S values, crucial for further scaling approaches.

### 3.5. Scaling Experiments: Exposure as Critical Factor

**Figure 1:**
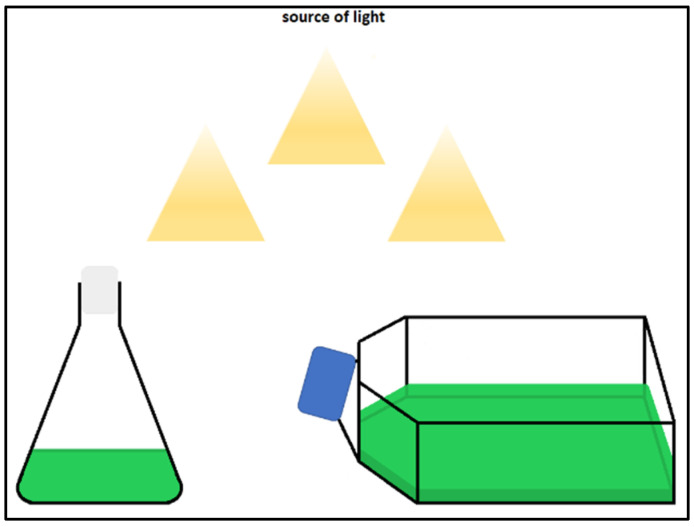
Culturing of cyanobacteria (flask, bottle): different incident light surfaces.

Photobiocatalysts are light-dependent organisms; so, the exposure is crucial for their metabolic activity. Scaling experiments were performed in correlation with different incident light surfaces (Figure 1), as an essential parameter for cyanobacteria to survive under non-physiological conditions, at the xenobiotic presence. The increase in scale was achieved with the changing of the substrate concentration from 1 to 10 mM, simultaneously in a flat bottle (better access to light) and in a culturing flask (Figure 1). Two strains of different activities towards ester bond hydrolysis, different selectivities, and of very different durations of reaction (3 versus 17 hrs) (results in Table 7) were selected for this experiment: the average: *Synechococcus bigranulatus* (CCALA 187), and the best: *Nostoc cf-muscorum* (CCALA 129). Table 8 shows the results of the hydrolysis conducted with *Synechococcus bigranulatus* (CCALA 187) in correlation to the exposure intensity.

The scale of the reaction was increased 10 times, compared to the previous research, and the results were as follows: 55% conversion degree, S = 21, and up to 86% of optical purity of received (*R*)-alcohol, achieved under limited light exposure in the flask. The degree of substrate conversion, in every case (Table 8) was higher in the bottle than in the flask. 

Studies carried out for the *Nostoc cf-muscorum* (CCALA 129) strain (Table 9) show another regularity: the degrees of conversion are at a similar level, regardless of the breeding method. However, despite this, the selectivity (S) and enantiomeric excesses strongly differed for particular tests (Table 9). Thus, in case of the *Nostoc cf-muscorum* strain (CCALA 129), the increase in the light exposure was followed by obtaining the pure alcohol: 1-(*R*)-phenylethanol of excellent enantiomeric excess up to ee> 99% for the substrate concentration range between 2mM, 4mM, and 6mM. However, this scaling approach was accompanied by a lowering in the conversion degree (to 7%), recompensated by the excellent selectivity (S = 275). In general, high values of enantioselectivities are related to the tests with a higher exposure (bottle reactions). Another aspect of *Nostoc cf-muscorum* (CCALA 129) application should be discussed; the scaling is also in correlation with the decrease in the optical purity of the unreacted substrate mixture from 96% of (*S*) ester (1mM experiments, Table 9) to 4% for 6 mM of substrate concentration. 

### 3.6. Viability of the Photobiocatalyst in the Presence of 1-Phenylethyl Acetate

Studies were carried out with the use of *Synechococcus bigranulatus* (CCALA 187), selected for flow cytometry analysis. The results are presented in Table 10. The reaction was carried out in culture bottles according to the general procedure.

The critical concentration, as can be seen from the above data, is 50mM of substrate concentration in the culture, which is lethal for most of the biocatalyst’s cells. Concentrations from 1 to 10mM are nearly neutral. 

## 4. Discussion on Photobiocatalytic Hydrolysis

The screening tests successfully identified biocatalysts with hydrolytic activity towards the substrate, a racemic mixture of 1-phenylethyl acetate (Table 4). These initial experiments also revealed the potential for side product formation under laboratory conditions. Specifically, the desired product of ester bond hydrolysis, phenylethyl alcohol, underwent oxidation to produce acetophenone. The rate of this side oxidation varied among the tested strains, which directly correlated with their differing requirements for replenishing the reducing components crucial for oxidoreductase activity. This can be disrupted in cultures maintained under non-physiological conditions for extended periods, a common occurrence in research settings. Cyanobacterial redox balance, particularly during the light phase of photosynthesis, depends on water accessibility and specific enzyme activities. However, these systems can sometimes fail or operate at suboptimal efficiency. Nevertheless, these metabolically flexible organisms can compensate for a lack of reducing power by utilizing protons and electrons from exogenous sources, such as alternative redox enzymes or, in these studies, phenylethyl alcohol. In such a context, hydrolysis of phenyl acetate is the first step of converting the substrate into the simpler accessible form for cyanobacterial cells, products, which are then incorporated in the redox system, crucial for the cells and related to the light regime. In addition, recognizing the side oxidation was critical for optimizing the duration of the biotransformation. Experiments exploring different process durations (Table 7) led to the conclusion that efficient hydrolysis via photobioconversion is a relatively short process, ideally completed within a maximum of 24 h. This parameter is typically sensitive for kinetically controlled reactions. Laboratory-scale experiments (1 mM substrate concentration) allowed for the identification of the most active photobiocatalysts. Significantly, the selectivity (S) values were good for whole-cell bioconversions (Table 7), and the reaction times were reasonable, up to 17 h. This was achieved for *Leptolyngbya foveolarum* (CCALA 76) and *Nostoc cf-muscorum* (CCALA 129).

Scaling Experiments and the Role of Light Exposure. The next research phase focused on scaling up the process. To obtain the most informative results, we compared the activity of the selected biocatalysts in relation to the exposure intensity, a parameter identified as critical for the survival of photo-dependent organisms under stress, such as the presence of xenobiotics. As previously noted (results in Table 7), *Synechococcus bigranulatus* (CCALA 187) and *Nostoc cf-muscorum* (CCALA 129) were evaluated for scaling purposes (Table 8 and Table 9). Interestingly, varying light availability led to entirely contrasting results for the tested strains. For *Synechococcus bigranulatus* (CCALA 187), reducing the exposure unexpectedly improved its biocatalytic parameters (Table 8). This led to an increase in selectivity (S) from 8.3 to 21, while maintaining the conversion degree at 55%, and increasing the enantiomeric excess (ee) of the (*R*)-alcohol from 68% to 86%, a significant improvement for bioconversion. Conversely, for *Nostoc cf-muscorum* (CCALA 129), different trends were observed (Table 9). Here, increasing the exposure significantly improved the optical purity (up to 99% of (*R*)-alcohol) of the product mixture and the electivity of the process (up to 275, Table 9). However, the conversion degrees dropped dramatically after the substrate concentration was merely doubled.

Implications for Future Research. These findings suggest that the selection of photocatalysts should be broadened to include experiments with different light exposures, even for strains whose activity towards the tested substrate initially appears average after simple screening. Furthermore, as with other biocatalyzed processes, the light intensity and its incident surface should be individually tested and adjusted for each specific strain and reaction. This is crucial due to light′s pervasive influence on various sectors of the proteome, acting as a strong and dominant factor in controlling gene expression, as demonstrated, for example, in *Synechocystis* sp. *PCC6803* [21]. Lighting conditions impact not only the growth rate but also the overall physiology of cyanobacterial cells [22]. There are reports for other *Synechococcus* sp. strains indicating that natural changes in light intensity significantly affect the expression of hundreds of genes controlled by the circadian clock. Moreover, under unnatural conditions of continuous irradiation, genes are activated differently than in the natural circadian cycle, thus confirming the significant impact of light intensity on their expression [23]. Considering the investigated effect of the incident light surface and intensity on the culture and the biotransformation process, it can be concluded that at least two crucial parameters are closely connected to these factors: the activity of hydrolytic enzymes, manifested as the conversion degree, and more importantly, the selectivity (S) of the reaction (Table 8 and Table 9).

## 5. Conclusions

In conclusion, adjusting the essential parameter of the kinetically controlled conversion, the reaction duration, allowed us to identify *Leptolyngbya foveolarum* (CCALA 76), *Nostoc cf-muscorum* (CCALA 129), and *Synechococcus bigranulatus* (CCALA 187) as strains capable of forming 1-(*R*)-phenylethanol with enantiomeric excesses of 89%, 88%, and 86%, respectively, at conversion degrees close to 50%. Simultaneously, an optically enriched mixture of the unreacted substrate, 1-(*S*)-phenylethyl acetate, was obtained; for instance, with *Leptolyngbya foveolarum* (CCALA 76), the enantiomeric excess reached up to 98% (Table 6 and Table 7). Unfortunately, these initial bioreactions exhibited unsatisfactory selectivity (S).

A significant improvement in selectivity was achieved by recognizing light exposure intensity as a critical factor influencing hydrolase activity. These efforts resulted in excellent selectivity S values: up to 283 for the formation of 1-(*R*)-phenylethanol with ee > 99% when assisted by the *Nostoc cf-muscorum* strain (CCALA 129) and increased incident light surface (Table 9), though this came with low conversion degrees. In contrast, for *Synechococcus bigranulatus*, a decreased incident light surface yielded the best results in scaling experiments (substrate concentration increased to 10 mM). This process achieved a 55% conversion degree and produced 1-(*R*)-phenylethanol with 86% ee and an E value of 21 (Table 8). This research aimed to examine the catalytic and hydrolytic potential of cyanobacteria, utilizing wild-type whole-cell biocatalysts to conduct reactions in the most economical and straightforward manner possible. The studies using the model substrate, racemic 1-phenylethyl acetate, have demonstrated that cyanobacterial enzymatic systems, including carboxylic ester hydrolases (CEHs, EC 3.1.1), are active towards xenobiotic substrates. This work can serve as a starting point for preparing chiral alcohols using these biocatalysts. Concurrently, such an approach can also yield biomass that can be utilized according to the principles of sustainable development after the bioconversions are completed. Thus, the novelty of these studies lies in the application of selected photobiocatalysts, the successful scaling up of the process, and the viability assessment aimed at evaluating their further usability.

## Figures and Tables

**Table 1:** Linearity.

Analyte	r	a	b	t_a_	t_b_
Acetophenone	0.999740	93439	−41280	141.2	−1.85
1-(*S*)-phenylethyl acetate	0.999503	381353	−10425	102.2	−0.71
1-(*R*)-phenylethyl acetate	0.999479	378964	−7945	99.8	−0.53
1-(*S*)-phenylethanol	0.998469	327894	−17308	58.2	−0.78
1-(*R*)-phenylethanol	0.999831	416429	−9310	175.2	−1.00

**Table 2:** System suitability tests and injection precision.

Analyte	Capacity Factor (k′)	Resolution (Rs)	Tailing Factor 10%	Theoretical Plate Number (N)	RSD (%)
acetophenone	1.2	8.5	1.0	61577	0.68
1-(*S*)-phenylethyl acetate	1.6	4.5	1.2	47133	0.54
1-(*R*)-phenylethyl acetate	1.7	2.6	1.6	36800	0.54
1-(*R*)-phenylethanol	2.2	6.0	3.0	17342	0.62
1-(*S*)-phenylethanol	2.3	1.8	2.4	14657	0.42

**Table 3:** Accuracy and intermediate precision.

				CV (%)
Analyte	Concentration [mM]	Recovery Coefficient (%)	The Same Day	Different Days and Different Operators
	8.56	98.9	0.71	0.65
acetophenone	25.67	102.8	0.34	1.26
	42.78	98.2	0.30	0.30
1-(*S*)-phenylethyl acetate	3.22	95.5	0.58	0.94
	9.67	100.9	0.66	0.56
	16.11	102.8	1.24	1.06
1-(*R*)-phenylethyl acetate	3.22	95.2	0.64	0.85
	9.67	101.0	0.67	0.58
	16.11	102.9	1.25	1.07
1-(*R*)-phenylethanol	4.14	95.2	0.70	1.15
	12.43	101.1	0.57	0.54
	20.71	100.3	2.18	1.79
1-(*S*)-phenylethanol	4.14	100.3	1.10	1.04
	12.43	101.7	0.79	0.92
	20.71	99.8	2.66	2.28

**Table 4:** Results of screening tests with cyanobacteria and racemic mixture of 1-phenylethyl acetate. Selectivity factor (S) values.

Photobiocatalyst		Enantiomeric Excess (%)		
Three Hours	Conversion Degree (%)	1-(*R*)-phenylethanol	1-(*S*)-phenylethyl Acetate	S
*Synechococcus bigranulatus* (CCALA 187)	53	63	50	7.1
*Nostoc cf-muscorum* (CCALA 129)	27	67	24	6.4
*Nodularia sphaerocarpa* (CCALA 114)	42	69	46	8.5
*Leptolyngbya foveolarum* (CCALA 76)	19	52	8	3.5
*Kamptonema animale* (CCALA 138)	13	31	<5	-
*Limnospira maxima* (CCALA 27)	33	35	11	2.3
**1 day**				
*Synechococcus bigranulatus* (CCALA 187)	89	31	91	5.1
*Nostoc cf-muscorum* (CCALA 129)	74	60	>99	19
*Nodularia sphaerocarpa* (CCALA 114)	80	35	80	4.6
*Leptolyngbya foveolarum* (CCALA 76)	74	59	87	10
*Kamptonema animale* (CCALA 138)	56	33	18	2.3
*Limnospira maxima* (CCALA 27)	95	19	62	2.4
**Three days**				
*Synechococcus bigranulatus* (CCALA 187)	>99	11	-	-
*Nostoc cf-muscorum* (CCALA 129)	96	22	97	5.2
*Nodularia sphaerocarpa* (CCALA 114)	>99	10	-	-
*Leptolyngbya foveolarum* (CCALA 76)	>99	29	-	-
*Kamptonema animale* (CCALA 138)	>99	10	-	-
*Limnospira maxima* (CCALA 27)	>99	29	-	-

**Table 5:** Acetophenone obtained during biotransformations of 1-phenylethyl acetate: screening tests.

Photobiocatalyst	Time (h)	Conversion (%)
*Synechococcus bigranulatus* (CCALA 187)	3	0
	24	0
	72	3.9
*Nostoc cf-muscorum* (CCALA 129)	3	0
	24	0
	72	2.0
*Nodularia sphaerocarpa* (CCALA 114)	3	1.2
	24	6.2
	72	12.2
*Leptolyngbya foveolarum* (CCALA 76)	3	1.0
	24	3.5
	72	12.0
*Kamptonema animale* (CCALA 138)	3	07
	24	2.0
	72	4.2
*Limnospira maxima* (CCALA 27)	3	0.2
	24	3.3
	72	20.2

**Table 6:** Biotransformations of 1-phenylethyl acetate by *Leptolyngba faveolarum* (CCALA 76) strain, including the oxidation of phenylethyl alcohol **.

	Conversion (%)		Enantiomeric Excess (%)	
Day	Reaction 1 *	Reaction 2 **	1-(*S*)-phenylethyl Acetate	1-(*R*)-phenylethanol
1	73	1.6	98	39
2	89	3.5	98	19
3	>99	15.6	>99	16
4	>99	16.0	-	21
8	>99	19.0	-	27
10	>99	22.0	-	35

* ester hydrolysis to alcohol; ** oxidation of alcohol to acetophenone.

**Table 7:** Dependence of selectivity (S) and optical purity of the products on the duration of the biotransformation process.

	(Hour)	Enantiomeric Excess (%)		(%)	
Photobiocatalyst	Time	1-(*R*)-phenylethanol	1-(*S*)-phenylethyl Acetate	Conversion	S
*Leptolyngbya foveolarum* (CCALA 76)	7	89	55	48	30
*Nostoc cf-muscorum* (CCALA 129)	17	88	71	53	33
*Nodularia moravica* (CCALA 797)	16	5	24	56	1.3
*Kamptonema animale* (CCALA 138)	23	18	39	53	2
*Synechococcus bigranulatus* (CCALA 187)	3	65	58	56	8.3
*Limnospira maxima* (CCALA 27)	5	25	30	54	2.2
*Nodularia sphaerocarpa* (CCALA 114)	8	63	47	52	7

**Table 8:** Influence of different breeding methods and substrate concentrations for strain *Synechococcus bigranulatus* (CCALA 187) on bioconversion of 1-phenylethyl acetate. Selectivity factor (S) values.

				Enantiomeric Excess (%)		
Culture Method	Time (Hour)	Final Concentration of Substrate (mM)	Conversion (%)	1-(*R*)-phenylethanol	1-(*S*)-phenylethyl Acetate	S
bottle	3	1	70	32	71	3.8
Erlenmeyer flask	3	1	51	53	52	5.4
bottle	7	2	74	47	74	5.8
Erlenmeyer flask	7	2	48	39	24	2.8
bottle	7	4	54	19	24	1.8
Erlenmeyer flask	7	4	34	45	24	3.3
bottle	10	10	58	37	36	3
Erlenmeyer flask	10	10	55	86	42	21

**Table 9:** Influence of different breeding methods for strain *Nostoc cf-muscorum* (CCALA 129) on bioconversion of 1-phenylethyl acetate. Selectivity factor (S) values.

				Enantiomeric Excess (%)		
Culture Method	Time (Hour)	Final Concentration of Substrate (mM)	Conversion (%)	1-(*R*)-phenylethanol	1-(*S*)-phenylethyl Acetate	S
bottle	9	1	12	52	9	3.4
Erlenmeyer flask	9	1	11	54	8	3.6
bottle	17	1	83	42	96	8.4
Erlenmeyer flask	17	1	79	45	92	8
bottle	10	2	18	99	15	165
Erlenmeyer flask	10	2	13	54	10	3.6
bottle	11	4	12	99	8	275
Erlenmeyer flask	11	4	10	53	7	3.5
bottle	12	6	7	99	4	283
Erlenmeyer flask	12	6	9	59	5	4
bottle	13	8	10	57	5	3.8
Erlenmeyer flask	13	8	6	51	3	3.2
bottle	15	10	7	53	3	3.4
Erlenmeyer flask	15	10	3	49	1	2.9

**Table 10:** Vitality of biocatalyst depending on the concentration of xenobiotic (1 to 50 mM) and duration of the exposition.

			(%)	
Biocatalyst	Time (Hour)	Final Concentration of Substrate (mM)	Live Cells	Dead Cells
*Synechococcus bigranulatus* (CCALA 187)	7	1	99	1.0
	7	2	97	3.0
	16	2	96	4.0
	16	4	90	10
	16	6	87	13
	16	8	85	15
	16	10	80	20
	10	50	30	70

## Data Availability

The original contributions presented in this study are included in the article. Further inquiries can be directed to the corresponding authors.
